# The Mysterious Risk of Arterial Thrombosis With COVID-19: A Case Series of Acute Limb Ischaemia in Vaccinated Patients

**DOI:** 10.7759/cureus.56425

**Published:** 2024-03-18

**Authors:** Isabelle Sanders, Philip Stather, Wissam Al-Jundi

**Affiliations:** 1 Vascular Surgery, Norfolk and Norwich University Hospitals NHS Foundation Trust, Norwich, GBR

**Keywords:** covid-19, case series, vaccinations, embolectomy, acute limb ischaemia, arterial thrombosis

## Abstract

Introduction

Coronavirus-19 (COVID-19) plays a vital role in viral-induced hypercoagulability through the initiation of a cytokine storm. This mechanism has been found to predispose unvaccinated patients to systemic complications including arterial thrombosis (AT) with poor 30-day amputation-free survival rates. There remains, however, little understanding regarding the incidence in patients who have received a COVID-19 vaccination. This study aims to assess the incidence, management and outcomes of vaccinated patients with COVID-19 who develop thrombotic complications to reduce amputation and direct mortality.

Methods

The case notes of all emergency patients with COVID-19 referred to the vascular services in a tertiary referral centre between November 2021 and April 2022 were reviewed. Patients who were unvaccinated or admitted with stroke or coronary thrombosis were excluded. The study was undertaken to measure 30-day outcomes.

Results

Between November 2021 and April 2022, 167,290 people tested positive for COVID-19 in Norfolk. Thirty-one patients under the vascular service had COVID-19, of which, one patient was unvaccinated. Only one vaccinated patient was referred with AT and had a positive COVID-19 result two days after admission. Above-knee amputation was performed within 30 days and he survived. Seventeen percent of patients contracted COVID-19 during their hospital admission.

Conclusion

The incidence of acute limb ischaemia in vaccinated patients is low; however, the 30-day outcomes remain poor. Compared to unvaccinated patients, there was a significant reduction in the presentation of AT in vaccinated patients during that timeframe, despite a higher background number of COVID-19 cases. Therefore, vaccination may minimise the risk of AT.

## Introduction

Coronavirus-19 (COVID-19) has been shown to heighten the risk of vascular emergencies such as arterial thrombosis (AT) and acute limb ischaemia (ALI) in unvaccinated patients [1]. ALI impacts approximately 14 out of 10,000 people annually, and among these cases, AT is the most complex subtype to treat [2]. This mechanism seems to be related to a cytokine storm precipitating a hypercoagulable state [3,4].

The development of vaccinations against COVID-19 was rapid and brought a glimmer of hope to stop the deadly pandemic. By July 2021, the first dose was offered to all adults in the United Kingdom [5]. Within just three months, over 45 million individuals in the UK had received their second vaccination by November 2021 [6]. Vaccinations have proven effective at reducing cases of the wild-type COVID-19 virus, and therefore the thrombotic complications that come with the disease [7]. A study conducted by Ismail et al. reported vaccine effectiveness against hospitalisation of 92%, two weeks after the second dose [8]. This applies to both the AstraZeneca and Pfizer-BioNTech vaccines. Conversely, there have been reports of thrombosis as a side effect of the vaccines [9]. This is thought to be caused by soluble adenoviral protein spike variants, leading to endothelial dysfunction and, subsequently, thrombosis [10].

We previously conducted a retrospective case series and systematic review at the Norfolk and Norwich University Hospital Foundation Trust to look at the incidence and 30-day outcomes of AT in patients with COVID-19 prior to the introduction of vaccines [1]. The results showed that the estimated incidence of symptomatic AT was 0.03%; however, the 30-day mortality rate for patients with COVID-19 and AT was 60% with a 30-day amputation-free survival rate of 29%. A follow-up case series at the same hospital trust is vital to directly compare the differences in vaccinated patients.

In this situation, it is imperative to understand the outcome of vaccinated patients and determine if there is a difference in risk. There is currently limited published data exploring the vascular complications of COVID-19 in vaccinated patients. This study aims to assess the incidence, management and outcomes of vaccinated patients with COVID-19 who develop thrombotic complications to reduce amputation and direct mortality.

## Materials and methods

Study design

This study presents a retrospective, single-centre, consecutive case series, which was performed in accordance with the Preferred Reporting of Case Series in Surgery (PROCESS) guidelines [11].

Settings, time frames and participants

Data was collected from the Norfolk and Norwich University Hospital Foundation Trust, a tertiary referral centre with a vascular unit, in June 2022. The case notes of all emergency patients diagnosed with COVID-19 who were referred to the vascular services between November 2021 and April 2022 were extracted from hospital medical records and reviewed. This includes both outpatients referred from clinicians, as well as hospitalised patients. Polymerase chain reaction (PCR) swab was used as the standard diagnostic tool for confirming COVID-19 infection. The chosen timeline for data collection was selected as by November 2021, 45,731,565 UK individuals had received their second vaccine [6]. There were no restrictions regarding age, co-morbidities, gender or ethnicity.

This study was conducted in accordance with the principles outlined in the Declaration of Helsinki [12]. Informed consent was waived due to the retrospective nature of the study. To uphold patient confidentiality and privacy, all patient-identifiable information was anonymised, and the participants were referred to by their unique participant number/code. Data security measures were enforced, with all data held electronically and secured using a password-protected folder, shared only through the vascular S: drive. This was only accessible to staff in the vascular department. 

Outcomes

The selection of outcomes and variables was based on studies conducted previously on unvaccinated patients [1]. The study was undertaken to measure 30-day outcomes. The recorded variables included demographic characteristics, past medical history, date of admission, the reason for admission, date of positive COVID test result, date of vaccination, presence of symptoms, computed tomography angiography (CTA) findings, management, postoperative complications, mortality and cause of death. The reason for admission was categorised into COVID-19 and ALI. Exclusion criteria were applied to patients who were unvaccinated or admitted with stroke or coronary thrombosis. Management was categorised into anticoagulation, embolectomy, amputation and palliation.

Statistical analysis

Microsoft Excel (Microsoft, Redmond, WA) was used to analyse the data. This data was presented as percentages. Statistical analyses of comparative data were conducted between the present study and a previous publication focusing on unvaccinated patients within the same trust. A chi-squared (χ^2^) test and Fisher’s exact test were conducted to analyse categorical variables.

## Results

Vascular referrals

**Figure 1:**
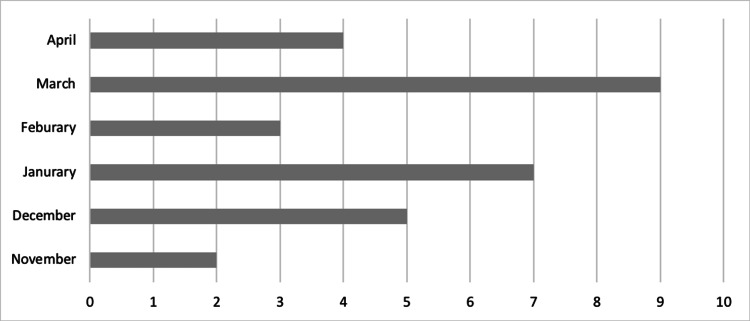
Number of referrals to the vascular services between November 2021 and April 2022.

Between November 2021 and April 2022, 31 patients were referred to the vascular services with a diagnosis of COVID-19. One of these cases was unvaccinated and therefore excluded from the study, totalling 30 patients. The majority of referrals occurred in March 2022 (9; 30%), January 2022 (7; 23%) and December 2021 (5; 17%) (Figure 1).

**Figure 2:**
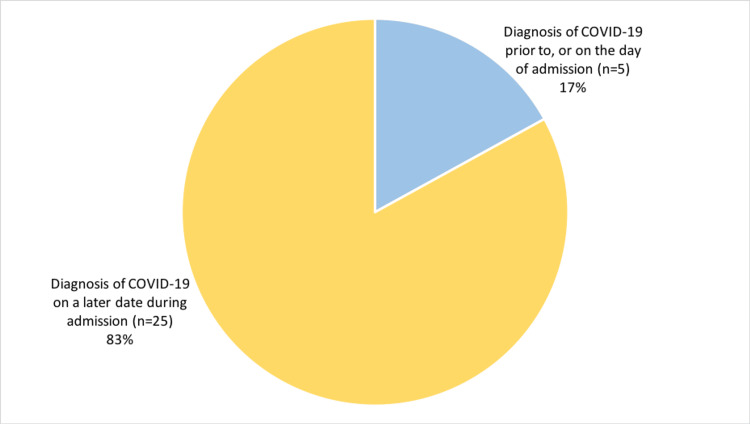
Date of COVID-19 test result classified into before or after admission, in patients referred to the vascular services between November 2021 and April 2022.

Out of the 30 cases, only five patients had a positive COVID-19 test prior to or on the day of admission. The remaining cases had a later diagnosis during their hospital stay (Figure 2). Within this six-month period, there were 167,290 cases of COVID-19 in Norfolk (catchment area of 1.1 m) [13].

Presentations

**Figure 3:**
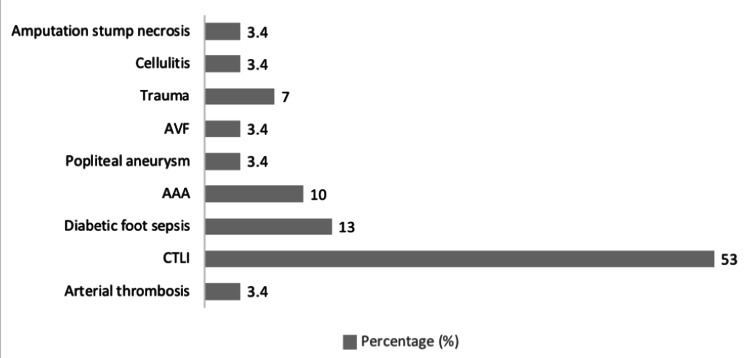
Patients with positive COVID-19 and reason for referral to the vascular services between November 2021 and April 2022. AVF, arteriovenous fistula; AAA, abdominal aortic aneurysm; CTLI, chronic threatening limb ischaemia.

Only one patient out of the 30 cases had a diagnosis of AT (3.4%). Other presentations consisted of chronic limb ischaemia (16; 53%), diabetic foot sepsis (4; 13%), abdominal aortic aneurysm (3; 10%), popliteal aneurysm (1; 3.4%), occluded arteriovenous fistula (1; 3.4%), trauma (2; 7%), cellulitis (1; 3.4%) and amputation stump necrosis (1; 3.4%) (Figure 3).

COVID-19 referrals

Out of the five patients who had a positive COVID-19 test prior to admission, there were four diagnoses of chronic threatening limb ischaemia (CTLI) and one abdominal aortic aneurysm. The majority of occlusions, identified by CTA, were located in the proximal limbs, including the superficial femoral and popliteal arteries. Three cases of CTLI were managed by surgery (one femoro-distal bypass and two amputations [left below-knee and toe]). The remaining case of CLI was treated by angioplasty.

Arterial thrombosis

Only one vascular patient with COVID-19 between November 2021 and April 2022 had a diagnosis of AT, specifically ALI. The positive PCR test was recorded two days after admission. Within 30 days, the patient was managed with an above-knee amputation (AKA) and survived.

Comparative analysis

To conduct a comparative analysis, the findings were compared to data from a previous case series on the incidence and outcomes of COVID-19 in unvaccinated patients within the same trust [1].

Incidence of AT

A chi-squared (χ^2^) test was conducted to analyse the incidence of AT during the six-month timeframe in vaccinated patients, compared to a 12-month timeframe in unvaccinated patients based on a previous study [1]. This resulted in p <0.00001; therefore, there was a significant difference in the incidence of AT between the two groups. Overall, patients who were not vaccinated were more likely to have AT than those who were vaccinated.

Demographics

A Fisher’s exact test was used to compare the number of comorbidities between the population in each case series. The results indicated that there was no significant difference (p = 0.9635).

Reason for Admission

Overall the number of admissions to the vascular service with a concurrent diagnosis of COVID-19 during both time frames was similar; however, the reason for admission varied. A Fisher’s exact test was used to compare the number of elective versus emergency cases in both cohorts. This revealed a higher proportion of elective cases in the cohort of vaccinated patients (p = 0.0014). In addition, the most common reason for admission changed from AT to CTLI. The total number of patients presenting with CTLI and COVID-19 in Norfolk was equivalent to 1/100,000 in the unvaccinated cohort (cohort A) and 3.5/100,000 in the vaccinated cohort (cohort B) (relative risk = 0.29; 95% CI: 0.06-1.38, p = 0.1182). The total number of patients with AT was equivalent to 1.5/100,000 in cohort A and 0.1/100,000 in cohort B (relative risk = 15; 95% CI: 1.98-113.56, p = 0.0087). The total number with AAA was equivalent to 0.3/100,000 in both cohorts (relative risk = 1; 95% CI: 0.20-4.95, p = 1.00).

Outcomes

Due to the variation in types of referrals, it is difficult to compare outcomes; however, mortality across the whole cohort in cohort A was 42% and in cohort B was 30%. Although there was a reduction in mortality for vascular patients diagnosed with COVID-19, this was not significant (p = 0.31).

## Discussion

This case series demonstrates that vaccinated patients have a reduced incidence of developing AT as a complication of COVID-19, shown by the clear reduction in the number of admissions compared to previous data on unvaccinated patients. However, the single case of AT consequently received an AKA, showing that the 30-day outcomes remain poor. To our knowledge, there has only been one other study comparing the incidence of ALI in vaccinated and unvaccinated patients [14]. Bowen Xie et al. observed that COVID-19-associated limb ischaemia predominately affected non-vaccinated patients, accounting for 92% of cases.

In COVID-19, vascular inflammation and stenosis arise from pathophysiological processes influenced by the infections' impact on the activation of platelets, hypercoagulability, stasis in prolonged immobilisation and vascular endothelial dysfunction [15]. Several authors have discussed the hypothesis that COVID-19 triggers a pro-inflammatory cytokine reaction, coupled with an upregulation in angiotensin-converting enzyme 2 (ACE2) expression, resulting in endothelial cell damage and, subsequently, thrombosis [16-18].

Various patient characteristics have been demonstrated to heighten the occurrence of coagulopathies among individuals with COVID-19. Reports indicate that the elderly population experiences more severe cases of COVID-19, with a higher prevalence of thrombosis [19,20]. Additionally, medications, including ACE inhibitors, angiotensin receptor blockers, thiazolidinediones and ibuprofen, have been shown to increase the incidence of thrombosis in COVID-19 due to the upregulation of ACE2. Consequently, individuals with co-morbidities requiring these medications for treatment, such as hypertension, diabetes, chronic kidney disease and cardiovascular disease, are at a heightened risk of developing more severe complications from COVID-19 [21].

Various presentations of thrombotic involvement in COVID-19 have been observed in reports, ranging from microvascular dysfunction to large-vessel thrombosis. A case series conducted by Poissy et al. revealed a significant increase in the occurrence of pulmonary embolism (PE) among COVID-19 patients in the intensive care unit (ICU), compared to 7.5% among those admitted for influenza [22]. A case series indicated that PE is among the most prevalent presentations of COVID-19-associated coagulopathy, along with venous thrombosis, bowel infarction and cerebral parenchymal infarction [23]. It has been shown that vaccine effectiveness against hospitalisation was 92%, two weeks after the second dose [8]. This reduction in cases further diminishes the thrombotic complications associated with the disease.

The previously published case series and systematic review of unvaccinated patients reports outcomes during the first year of the pandemic prior to the availability and offering of the COVID-19 vaccination programme and the emergence of subsequent variants of the coronavirus [1]. Hence, the results from this previous study may not be applicable to the more recent dominant variants, e.g., Omicron. Therefore, this follow-up series on vaccinated patients was conducted. These results highlight a few differences. The incidence of AT with COVID-19 was significantly reduced, as only one patient was referred to the vascular services with this diagnosis during this six-month period. As the diagnosis of COVID-19 was confirmed two days after admission, it can be debated as to whether the thrombus was caused as a complication of the virus or caught as an in-patient. This would only be possible to comment on if a PCR test was conducted prior to admission; however, studies have shown that patients can become positive after more than two days of coming into contact with the virus [24]. Additionally, there were no other cases of COVID-19 on the ward at that time to spread the virus to this patient.

Another comparison between the two case series was the number of vascular referrals. A similar number of patients were referred to the vascular services within half the time in the follow-up series (six months) compared to the first series (12 months). However, during the follow-up series, there were significantly more cases in the community with a lower proportion of them presented at the hospital. In addition, a high proportion contracted COVID-19 whilst already being an inpatient under the vascular service rather than being admitted due to COVID-19. Proportionally, the number of referrals was reduced. Overall, the demographics of both cohorts were similar and the incidence of AAA remained constant. Despite the increase in the number of patients with COVID-19, there was a reduction in AT in the vaccinated group. However, there was an increase in CTLI cases. CTLI patients may have been more reluctant to or advised not to attend hospital during 2020-2021.

The main limitation of this study was the significant difference in the study duration between the previous data on unvaccinated patients and this case series, making it difficult to directly compare the results. As there was only a single case of AT in this case series, a longer duration of follow-up may have highlighted more cases which would enable more in-depth analysis.

## Conclusions

The incidence of ALI in vaccinated patients is low; however, the 30-day outcomes remain poor. More research over a longer period is required to directly compare the incidence of ALI between vaccinated and non-vaccinated patients; however, there was a significant reduction in presentation of AT in vaccinated patients during that timeframe, despite a higher background number of COVID-19 cases. Therefore, vaccination may minimise the risk of AT.
